# Renoprotective Effects of DPP-4 Inhibitors

**DOI:** 10.3390/antiox10020246

**Published:** 2021-02-05

**Authors:** Daiji Kawanami, Yuichi Takashi, Hiroyuki Takahashi, Ryoko Motonaga, Makito Tanabe

**Affiliations:** Department of Endocrinology and Diabetes Mellitus, Fukuoka University School of Medicine, Fukuoka 814-0180, Japan; y.takashi.si@fukuoka-u.ac.jp (Y.T.); htakahashi@fukuoka-u.ac.jp (H.T.); ryoko-na@adm.fukuoka-u.ac.jp (R.M.); mtanabe@live.jp (M.T.)

**Keywords:** DPP-4, DPP-4 inhibitors, diabetic kidney disease, diabetic nephropathy

## Abstract

Diabetic kidney disease (DKD) is the leading cause of end-stage renal disease (ESRD) worldwide. Dipeptidyl peptidase (DPP)-4 inhibitors are widely used in the treatment of patients with type 2 diabetes (T2D). DPP-4 inhibitors reduce glucose levels by inhibiting degradation of incretins. DPP-4 is a ubiquitous protein with exopeptidase activity that exists in cell membrane-bound and soluble forms. It has been shown that an increased renal DPP-4 activity is associated with the development of DKD. A series of clinical and experimental studies showed that DPP-4 inhibitors have beneficial effects on DKD, independent of their glucose-lowering abilities, which are mediated by anti-fibrotic, anti-inflammatory, and anti-oxidative stress properties. In this review article, we highlight the current understanding of the clinical efficacy and the mechanisms underlying renoprotection by DPP-4 inhibitors under diabetic conditions.

## 1. Introduction

The number of patients with type 2 diabetes (T2D) is increasing worldwide. Strict glycemic control has been shown to prevent the onset or progression of diabetic complications, including diabetic kidney disease (DKD). It is well-established that hyperglycemia triggers oxidative stress and the inflammatory process in the kidney. As such, it is ideal to administer anti-hyperglycemic agents with anti-oxidative stress and anti-inflammation properties. Dipeptidyl peptidase (DPP)-4 inhibitors are a class of oral glucose-lowering drugs that increase insulin secretion and decrease glucagon secretion by preventing the degradation of incretins. DPP-4 inhibitors are known to have a low hypoglycemic risk and weight-neutral effects. Furthermore, saxagliptin, sitagliptin, alogliptin, and linagliptin have been evaluated for their cardiovascular (CV) safety in randomized clinical trials (RCTs). However, while these studies revealed the CV safety of DPP-4 inhibitors, results are conflicting regarding the renal outcomes.

DKD is a leading cause of end-stage renal disease (ESRD). DKD is characterized by albuminuria and a reduction in the estimated glomerular filtration rate (eGFR). It is widely recognized that early intervention for multiple risk factors, including hyperglycemia, hypertension, and dyslipidemia, is important for preventing onset and progression of DKD. Clinically, renin-angiotensin aldosterone system (RAAS) inhibitors have been used to manage DKD because of their anti-albuminuric effects. Recent large-scale RCTs have shown that sodium glucose cotransporter 2 (SGLT2) inhibitors and glucagon-like peptide (GLP)-1 receptor agonists (GLP-1RAs) have favorable effects on CV and renal outcomes. Based on these findings, the use of these drugs has been suggested for T2D patients with CV risk factors. However, these drugs may exert different renoprotective effects, and how to use them properly remains controversial.

Benefits of DPP-4inhibitor include the fact that they are oral agents and can be administered to T2D patients with an impaired renal function who are receiving hemodialysis. The present review article summarizes our current understanding of the efficacy of DPP-4 inhibitors on DKD from both a basic and clinical standpoint and reconsiders the utility of DPP-4 inhibitors from a renoprotective perspective.

## 2. DPP-4 in Kidney

DPP-4 (also known as CD26) is serine peptidase that exists in various organs including kidney [1,2]. DPP-4 is expressed as a type II transmembrane glycoprotein, with a short 6-amino acid cytoplasmic tail and is active as a dimer with a monomer molecular weight of 110 kDa [3]. It consists of a large extracellular domain, anchored to the cell membrane by a flexible segment coupled to a trans-membrane sequence, with a short intracellular tail at the *N*-terminus. It is cleaved at the stalk to release the extracellular domain, which circulates in the plasma as soluble form (aa 49-766) [4]. Both the membrane-bound and soluble isoforms have catalytic activity, selectively removing *N*-terminal dipeptide from peptides with proline or alanine (with a greater affinity for proline than alanine) [2]. DPP-4 is responsible for the degradation of incretins. Glucose-dependent insulinotropic polypeptide (GIP) and GLP-1 are the two primary incretin hormones that are released from the small intestine in response to the ingestion of various nutrients. Under physiological conditions, the half-life of GLP-1 and GIP is 1–2 min because they are promptly degraded by DPP-4. They stimulate cAMP-dependent protein kinase A (PKA) activation through the receptors of GLP-1 and GIP in pancreatic β cells, thereby promoting insulin secretion with little to no effect on insulin secretion under low glucose conditions [5,6].

Previous studies have demonstrated that DPP-4 correlates with adipocyte inflammation and insulin resistance [7,8]. However, a recent study highlighted the complexity of DPP-4 activities and soluble DPP-4 levels. Varin et al. reported that adipocyte DPP-4 contributes to circulating soluble DPP-4 levels but not to glucose homeostasis [9]. However, they also found that hepatocyte DPP-4 contributes to its circulating activity and hepatic/adipose inflammation, and soluble DPP-4 is induced by systemic DPP-4 enzymatic inhibition [9], indicating that the DPP-4 activity and soluble DPP-4 levels do not always perfectly reflect metabolic inflammation.

In the kidney, DPP-4 has been shown to localize in the brush border of the membrane of the renal proximal tubules (segments 1–3), Henle’s loop, distal tubules, collecting ducts, glomerular visceral epithelial cells, and endothelial cells [10,11,12]. GLP-1R has been shown to be expressed in both the renal cortex and the proximal tubules [13,14] but several reports showed absence of GLP-1R in tubules [15,16,17]. Investigations by use of monoclonal antibodies against GLP-1R have revealed that it expressed mainly in the vasculature of the kidney [15,16,18]. Taken together, the presence of GLP-1R in the renal vasculature has been confirmed, but not in the tubules [19].

The DPP-4 activity and soluble DPP-4 levels have been extensively investigated to determine their utility as potential clinical markers of DKD. Duvnjak et al. reported that the serum DPP-4 activity is positively correlated with albuminuria [20]. A cross-sectional study of 1193 newly diagnosed T2D patients showed that a higher serum DPP-4 activity was associated with the presence of albuminuria and an impaired renal function (eGFR <60 mL/min/1.73 m^2^) [21]. A prospective study including 664 T2D patients showed that the baseline DPP-4 activity was an independent predictor of an increase in inflammatory markers and the urinary albumin-creatinine ratio (UACR) over a 4-year period, suggesting that the serum DPP-4 activity is an important predictor of the onset of DKD [22]. In addition, elevated serum soluble DPP-4 levels have been shown to be associated with a decreased renal function in T2D patients [23]. Recently, Baggio et al. showed that a prolonged DPP-4 inhibition increases the soluble DPP-4 levels in lymphocyte-enriched organs in mice. They noted that systemic DPP-4 inhibition increased the plasma levels of inflammatory markers in regular chow-fed but not in high-fat-diet-fed mice. However, they also found that soluble DPP-4 and inflammation marker levels were reduced in metformin-treated patients with T2D and cardiovascular disease (CVD) [24]. The significance of the serum DPP-4 activity and soluble DPP-4 levels in DKD remains unclear.

## 3. Mechanisms Underlying Renoprotection by DPP-4 Inhibitors

**Figure 1:**
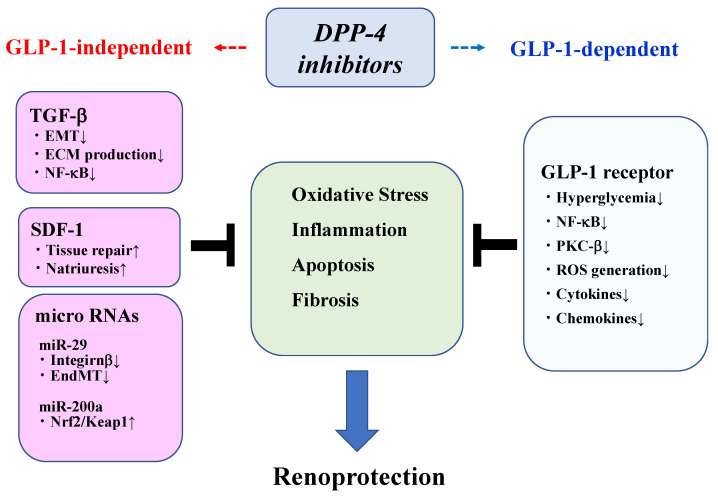
Renoprotective mechanisms of DPP-4 inhibitors. DPP-4 inhibitors exert renoprotection in both GLP-1-dependent and GLP-1-independent manners. The GLP-1-dependent pathway is mediated by GLP-1R. GLP-1-independent mechanisms are mediated by upregulating SDF-1 (DPP-4 substrate), micro RNAs such as miR-29 and miR-200a, and downregulating TGF-β signaling.

Inflammation, oxidative stress, and fibrosis play important roles in the development of DKD [25,26,27]. Oxidative stress and inflammatory signaling pathways promote the renal expression of transforming growth factor (TGF)-β and the production of extracellular matrix (ECM) via NF-κB activation [28], leading to glomerulosclerosis as well as tubulointerstitial fibrosis [29]. Epithelial-to-mesenchymal transition (EMT) is also implicated as a source of ECM production from fibroblasts in DKD [30]. Inflammatory cytokines, including tumor necrosis factor (TNF)-α, interleukin (IL)-1β, IL-6, and IL-18, induce the expression of chemokines, such as monocyte chemoattractant protein (MCP)-1, which promotes macrophage infiltration toward mesangial cells. Advance glycation end products (AGEs) activate several intracellular signaling cascades upon interaction with receptor for AGEs (RAGE), so the AGE-RAGE axis also plays a crucial role in the pathogenesis of DKD [31]. NOD-like receptor protein 3 (NLRP3) inflammasome has been shown to play an important role in the pathogenesis of DKD by promoting inflammation through the regulation of IL-1β and IL-18 production [32]. Podocyte injury/apoptosis plays a key role in the development of albuminuria [33]. Furthermore, endothelial-mesenchymal transition (EndMT), which alters the phenotype of endothelial cells into a mesenchymal-like phenotype, has been implicated in the pathogenesis of fibrosis in DKD [34,35]. A series of experimental studies has shown DPP-4 inhibitors to have beneficial effects on renal inflammation/oxidative stress, natriuresis, fibrosis, and apoptosis under diabetic conditions and to exert renoprotective effects in diabetic models as well as non-diabetic CKD models. Therefore, gut-renal axis has been proposed as a therapeutic target of incretin-based therapies [36]. Finally, modulation of innate immunity has been implicated in DPP-4 inhibitors-mediated renoprotection [37]. Interestingly, DPP-4 inhibitors can exert renoprotection in a GLP-1-depedent or independent fashion that are associated with DPP-4 substrates. The renoprotective effects of DPP-4 inhibitors are summarized in Figure 1.

### 3.1. Oxidative Stress/Inflammation

As described above, a series of experimental studies has shown that DPP-4 inhibitors attenuate DKD by inhibiting inflammation, oxidative stress, and fibrosis. GLP-1 has been shown to exert those renoprotective effects through GLPI-1R [38]. GLP-1 prevents renal oxidative stress by inhibiting NADPH oxidase through the activation of PKA and the production of cAMP. Recombinant human GLP-1 reduces PKC-β but increases protein PKA, which reduces oxidative stress in both glomeruli and tubules [39]. GLP-1 induces anti-inflammatory effects by downregulating reactive oxygen species (ROS) generation in vascular cells [40,41] and renal cells [42,43]. DPP-4 inhibitors have been shown attenuate ROS generation and increase superoxide dismutase (SOD) activity. Furthermore, accumulating evidence indicates that the transcription factor Nrf2 plays a pivotal role in regulating redox homeostasis by increasing antioxidant genes to maintain intracellular redox homeostasis. Nrf2 is known to stimulate the heme oxygenase-1 (HO-1) axis, which is a potent anti-inflammatory target [44]. It has been reported that DPP-4 inhibitors activate the Nrf2/HO-1 pathway [45,46,47]. In addition, Nrf2/Kelch-like ECH-associated protein1 (Keap1) signaling pathways play an important role in preventing oxidative stress [48]. Sitagliptin has been shown to attenuate DKD in Goto–Kakizaki diabetic rats by downregulating mi-R200a that inhibits Nrf2/Keap1 pathway [49]. Toll-like receptor (TLR) 4 is widely expressed in mesangial cells, tubular epithelial cells, and podocytes. TLR4 has been implicated in inflammatory process in the development of DKD. Upon activation by binding the released endogenous ligands in immune and kidney cells, TLR4 initiates the activation of NF-κB [50]. DPP-4 inhibitors have been shown to exert anti-inflammatory effects by modulating the TLR4/NF-kB pathway [51,52,53].

### 3.2. Natriuresis

GLP-1RAs stimulate diuresis and natriuresis via GLP-1R [38], which is associated with the inhibition of Na^+^/H^+^ exchanger 3 (NHE3) in the proximal tubules. NHE3 plays an important role in reabsorbing filtered Na^+^ in the proximal tubules [54]. Therefore, inactivation of NHE3 can result in natriuresis. However, GLP-1-indepdent pathways seem to be involved in DPP-4 inhibitor-mediated natriuresis [55,56]. Moroi et al. showed that teneligliptin can induce natriuresis in the presence of GLP-1R antagonist [57]. Furthermore, Lovshin et al. performed a RCT to investigate the effects of sitagliptin on urinary sodium excretion in T2D patients. They observed that one-month administration of sitagliptin significantly increased the total fractional sodium excretion compared with placebo [58]. This finding is associated with increased levels of intact stromal cell-derived factor (SDF)-1α, a DPP-4 substrate. Taken together, these findings suggest that DPP-4 inhibitors induce natriuresis in a GLP-1-independent manner.

### 3.3. Fibrosis

Modulation of EMT and EndMT programs is involved in the anti-fibrotic effects of DPP-4 inhibitors. Linagliptin has been shown to prevent pro-fibrotic EndMT by inducing miR-29, a microRNA that can disrupt the interaction between membrane-bound DPP-4 and integrin β1, which enhances TGF-β signaling and vascular endothelial growth factor receptor (VEGFR)1 [59,60]. Vildagliptin has been shown to attenuate liver fibrosis by downregulating ERK1/2, p38MAPK, and NF-κB signaling [61]. Another study showed that linagliptin attenuated high-fat diet (HFD) and streptozotocin (STZ)-induced liver fibrosis in rats by reducing levels of inflammatory mediators, such as TNF-α, IL-6, NF-κB, and collagen depositions, accompanied by inhibiting the expression of collagen, α-SMA, and TGF-β1 in the liver [62]. Several drugs have been shown to be involved in DPP-4 inhibition-associated anti-fibrotic responses in DKD [62]. Both angiotensin-converting enzyme inhibitors (ACEis) and angiotensin II receptor blockers (ARBs) are widely used to treat DKD because they have been shown to improve glomerular hypertension. Srivastava et al. showed that the ACEi imidapril, but not the ARB TA-606, attenuated renal fibrosis in STZ-induced diabetic CD-1 mice by inhibiting DPP-4 and TGF-β signaling [63]. From a mechanistic standpoint, they observed that ACEis protected against renal fibrosis by increasing endogenous levels of anti-fibrotic peptide *N*-acetyl-seryl-aspartyl-lysyl-proline (AcSDKP), a substrate of ACE, either by suppressing DPP-4-associated mesenchymal transformation or by elevating the gene expression of antifibrotic miR-29 [63]. Wahba et al. reported that vitamin D3 potentiated the anti-fibrotic effects of metformin in a rat model of metabolic syndrome via AMPK/SIRT1 activation and DPP-4 inhibition [64].

Aanagliptin has been shown to inhibit liver fibrosis and carcinogenesis in genetically obese melanocortin 4 receptor-deficient mice [65]. Mechanistically, anagliptin inhibited the inflammatory and fibrotic gene expression in macrophages [65]. Increased DPP-4 activity has been implicated in TGF-β-mediated fibroblast activation [66]. The effects of DPP-4 inhibitors on cardiac fibrosis have also been investigated. In addition, DPP-4 inhibitors are involved in cardiac fibrosis. Hirose et al. showed that the genetic deletion of DPP-4 and MK-0626, an analog of des-fluorositagliptin, attenuated cardiac fibrosis in a transverse arterial constriction-induced pressure overload heart failure mouse model [67]. Brown et al. found that saxagliptin attenuated angiotensin II-induced cardiac fibrosis in mice and proposed a potential mechanism whereby saxagliptin suppresses local inflammatory signaling by modulating cardiac inflammatory cascades via the attenuation of cardiac CD11c-expressing dendritic cells or the activation of immunosuppressant CD8+ lymphocytes [68].

## 4. DPP-4 Substrates

As mentioned before, a number of proteins other than incretins have been shown to be degraded by DPP-4. Physiological substrates include GIP, GLP-1, GLP-2, peptide tyrosine tyrosine (PYY), stromal cell-derived factor-1 (SDF-1), and substance P (SP), and pharmacological substrates include brain natriuretic peptide (BNP), erythropoietin, and neuropeptide Y (NPY). Whether or not the prevention of the degradation of these substrates by DPP-4 inhibitors is associated with the renoprotective effects of DPP-4 inhibitors is unclear at present. Below, we describe several substrates that are involved in the pathogenesis of DKD.

### 4.1. BNP

BNP is secreted from cardiomyocytes in response to volume expansion and pressure overload. BNP is clinically used for the diagnosis and evaluation of CVD [69]. BNP is synthesized as a 134-amino acid precursor (prepro BNP) that is processed to proBNP [1,2,3,4,5,6,7,8,9,10,11,12,13,14,15,16,17,18,19,20,21,22,23,24,25,26,27,28,29,30,31,32,33,34,35,36,37,38,39,40,41,42,43,44,45,46,47,48,49,50,51,52,53,54,55,56,57,58,59,60,61,62,63,64,65,66,67,68,69,70,71,72,73,74,75,76,77,78,79,80,81,82,83,84,85,86,87,88,89,90,91,92,93,94,95,96,97,98,99,100,101,102,103,104,105,106,107,108] and subsequently cleaved by furin or corin to BNP [1,2,3,4,5,6,7,8,9,10,11,12,13,14,15,16,17,18,19,20,21,22,23,24,25,26,27,28,29,30,31,32]. Experimental studies have shown that BNP is cleaved by DPP-4 to BNP [1,2,3,4,5,6,7,8,9,10,11,12,13,14,15,16,17,18,19,20,21,22,23,24,25,26,27,28,29,30,31,32] and BNP [3,4,5,6,7,8,9,10,11,12,13,14,15,16,17,18,19,20,21,22,23,24,25,26,27,28,29,30,31,32], the latter of which has less natriuretic, diuretic, and vasodilatory activity than BNP [1,2,3,4,5,6,7,8,9,10,11,12,13,14,15,16,17,18,19,20,21,22,23,24,25,26,27,28,29,30,31,32]. Several studies have suggested that elevated serum BNP levels may be a predictor for the progression or prognosis of DKD [70,71,72]. Thus far, however, no report has shown that increased BNP levels by DPP-4 inhibitors are associated with the pathogenesis of DKD.

### 4.2. Erythropoietin

Erythropoietin is regulated by hypoxia-inducible factor (HIF)-1 and implicated in hematopoiesis [73]. Erythropoietin may have direct renoprotective effects [74] and be involved in the renoprotection induced by SGLT2 inhibitors [75]. Erythropoietin has been shown to be cleaved by DPP-4. Although cleaved erythropoietin has been proposed to function as a competitive inhibitor for full-length erythropoietin [76], the clinical significance of these observations, particularly in DKD, remains unclear.

### 4.3. NPY

NPY is a hypothalamus-derived peptide that is widely distributed in the nervous system and localized in adipose tissues that are involved in energy metabolism [77]. Cleavage of native NPY [1,2,3,4,5,6,7,8,9,10,11,12,13,14,15,16,17,18,19,20,21,22,23,24,25,26,27,28,29,30,31,32,33,34,35,36] has been shown to generate NPY [3,4,5,6,7,8,9,10,11,12,13,14,15,16,17,18,19,20,21,22,23,24,25,26,27,28,29,30,31,32,33,34,35,36], which has strong affinity to NPY receptors NPYR2 and NPYR5 [2]. Recently, Lay et al. demonstrated a relationship between NPY and albuminuric kidney disease, including DKD [78]. In that study, they found that the glomerular NPY expression was increased in T2D patients with DKD. Interestingly, they also found a significant correlation between the glomerular NPY expression and eGFR. Consistent with these observations, NPY-deficient diabetic mice showed reduced albuminuria and were protected from podocyte damage compared to wild-type mice [78]. While the clinical significance of the cleavage of NPY by DPP-4 inhibitors remains unclear at present, the findings of further studies regarding this issue will be interesting.

### 4.4. SDF-1

SDF-1, also known as CXCL12, is a chemokine that is widely expressed in many organs. It comprises two isoforms: SDF-1α and SDF-1β. Both isoforms have been shown to be cleaved by DPP-4. SDF-1 is known to be involved in tissue repair by enhancing the migration of endothelial progenitor cells to sites of acute injury such as ischemia [79]. Conflicting results regarding the roles of SDF-1 in DKD have been reported. The inhibition of SDF-1 reportedly resulted in the attenuation of albuminuria in db/db mice [80]. In contrast, decreased endothelial SDF-1α was accompanied by proteinuria, enhanced oxidative stress, podocyte foot process effacement, and an increased glomerular size in Zucker obese rats, all findings that were prevented by linagliptin [81]. Another study showed that linagliptin attenuated the progression of albuminuria, glomerulosclerosis, periglomerular fibrosis, podocyte loss, and renal oxidative stress in GLP-1R-deficient diabetic mice, suggesting that linagliptin inhibits DKD in a SDF-1-dependent manner [82].

## 5. Preclinical Studies

### 5.1. Diabetic Animal Models

Liu et al. showed that the DPP-4 inhibitor LAF237 reduced albuminuria, interstitial expansion, glomerulosclerosis, and the thickening of the glomerular basement membrane in STZ-induced diabetic rats. From a mechanistic standpoint, LAF237 inhibited the DPP-4 activity and increased active GLP-1 levels, thereby preventing oxidative stress and renal cell apoptosis, leading to the downregulation of TGF-β1 [83]. Kodera et al. also showed that PKF275-055, a DPP-4 inhibitor, decreased urinary albumin excretion and ameliorated histological changes due to DKD in type 1 diabetes (T1D) rats independent of the glucose-lowering effects [84]. They also showed that PKF275-055 inhibited glomerular macrophage infiltration by suppressing the NF-κB activity [84]. Vildagliptin has been shown to restore the impaired myogenic contraction of renal arteries and increased expression of p22phox, an NADPH oxidase complex, in Zucker Diabetic Fatty (ZDF) rats independent of the glucose-lowering effects [85]. Birnbaum et al. showed that saxagliptin reduced the serum blood urea nitrogen and creatinine levels in BTBR (T2D) and Akita (T1D) mice, accompanied by reduced serum levels of C-reactive protein, TNF-α, IL-1β, IL-6, and IL-18 [86]. Nakashima et al. reported that linagliptin attenuates albuminuria in T1D rats by downregulating the AGE-RAGE axis independent of the glucose-lowering effects [87]. Linagliptin has been shown to inhibit tubular damage by attenuating oxidative stress, inflammation, and fibrosis in fructose-STZ (Fr-STZ)-induced diabetic rats, as proven by reductions in the levels of kidney injury molecule (KIM)-1 and neutrophil gelatinase-associated lipocalin (NGAL) [88]. Linagliptin has also been shown to attenuate albuminuria and kidney hypertrophy in a glucose-reduction-independent manner by upregulating renal antioxidants catalase and manganese superoxide dismutase (MnSOD) levels [89]. Finally, Takagaki et al. showed that teneligliptin ameliorated the DPP-4-mediated-EMT program in a bovine serum albumin (BSA)-induced kidney injury model [90]. These findings indicate that the administration of DPP-4 inhibitors results in the attenuation of renal inflammation and oxidative stress under diabetic conditions.

In addition to inflammation and oxidative stress, DPP-4 inhibitors prevent fibrotic process in diabetic mice. Sitagliptin has been shown to attenuate proteinuria, renal injury, and fibrosis by inhibiting the TGF-β/Smad pathway in STZ-induced DKD in a glucose-reduction-independent manner [91]. Similarly, sitagliptin attenuated renal tubulointerstitial fibrosis by inhibiting the Wnt/β-catenin signaling pathway [92]. Furthermore, saxagliptin inhibited tubulointerstitial fibrosis by attenuating renal hypertrophy, TGF-β-induced fibrosis, and NF-κB-mediated macrophage infiltration [93]. Kanasaki et al. showed that linagliptin ameliorates diabetic kidney fibrosis by EndMT, which is associated with the inhibition of the DPP-4 protein expression by miR-29 that negatively regulates the 3′ untranslated region (UTR) of DPP-4 mRNA [94]. Accordingly, Shi et al. reported that linagliptin, but not sitagliptin, inhibited the TGF-β2-induced EndMT and DPP-4 3′UTR activity in human dermal microvascular endothelial cells [95], indicating that the attenuation of EndMT by linagliptin is drug-specific effect. The difference in the effects of these DPP-4 inhibitors seems to be dependent on their ability to inhibit homo-dimer formation of DPP-4, which was observed only in linagliptin [95].

Linagliptin has been shown to reduce podocyte injury in a glucose-reduction-independent manner in T2D (db/db) mice [96]. Kubo et al. investigated the DPP-4 activities in human renal biopsy specimens of glomerulosclerosis, including DKD. They found that the DPP-4 activity was increased in podocytes with DKD. Accordingly, they found that adriamycin increased the DPP-4 activity in cultured podocytes, with this effect inhibited by saxaglptin. Furthermore, saxagliptin prevented podocyte damage by maintaining synaptopodin and RhoA, which are important components of the cytoskeleton structure of podocytes [97]. Sitagliptin has been shown to prevent apoptotic cell death in the kidney of ZDF-rats, as indicated by reductions in the BAX/Bcl-2 ratio, Bid protein levels, and numbers of terminal deoxynucleotidyl transferase dUTP nick-end labeling (TUNEL)-positive cells in the kidney sections [98]. These findings reveal that DPP-4 inhibitors attenuate DKD via anti-apoptotic effects.

### 5.2. Non-Diabetic CKD Models

Linagliptin has been shown to inhibit tubulointerstitial fibrosis and albuminuria in non-diabetic rats with 5/6 nephrectomy [99]. Consistently, the administration of linagliptin ameliorated gentamycin-induced renal injury and restored the renal functional, oxidative, inflammatory, apoptotic and histopathological changes [100]. Sitagliptin reduces inflammatory mediators, such as NF-κB, TNF-α, IL-1β, IL-6, and MCP-1, in the kidneys of a salt-dependent hypertension CKD model [101]. Mechanistically, sitagliptin promoted macrophage polarization toward the anti-inflammatory M2 phenotype [101]. Tanaka et al. showed that linagliptin and higher concentrations of sitagliptin, vildagliptin, and alogliptin inhibited the FFA-bound albumin-induced increases in mRNA expression of MCP-1 in cultured mouse proximal tubular cells [102]. Consistent with these observations, linagliptin significantly inhibited lipotoxicity-induced tubulointerstitial injury [102]. Linagliptin suppressed the induction of pro-fibrotic miRNAs, such as miR-199a-3p, and restored the levels of anti-fibrotic miR-29c in 5/6 nephrectomy rats [103].

### 5.3. GLP-1R-Deficient Mice

DPP-4 inhibitors have been shown to inhibit DKD in GLP-1R-deficient mice. Hasan et al. showed that linagliptin attenuated renal interstitial fibrosis by inhibiting TGF-β/Smad3-mediated fibrotic response in GLP-1R-deficient mice with 5/6 nephrectomy [104]. As mentioned above, Takashima et al. demonstrated that alogliptin ameliorates DKD by upregulating SDF-1α in GLP-1R-deficient diabetic-prone KK/Ta-Akita mice [82]. These findings indicated that DPP-4 inhibitors can attenuate DKD via a GLP-1-independent pathway.

## 6. Clinical Trials on Renal Outcomes

### 6.1. Linagliptin

The Cardiovascular and Renal Microvascular Outcome Study with Linagliptin (CARMELINA) enrolled 6991 T2D patients with a high CV risk (history of vascular disease and UACR > 200 mg/g) and high renal risk (reduced eGFR and micro- or macroalbuminuria). The primary outcome was a composite of CV death, nonfatal myocardial infarction, and nonfatal stroke (three-point major adverse cardiovascular events: 3P-MACE). The secondary outcome was the time to the first occurrence of renal death, ESRD, or a sustained ≥40% reduction in the eGFR from baseline. After a median follow-up of 2.2 years, 3P-MACE occurred in 434 of 3494 (12.4%) and 420 of 3485 (12.1%) subjects in the linagliptin and placebo groups, respectively (hazard ratio (HR) 1.02, 95% confidence interval (CI): 0.89–1.17). The composite renal outcome occurred in 327 of 3494 (9.4%) and 306 of 3485 (8.8%) subjects in the linagliptin and placebo groups, respectively (HR 1.04, 95% CI: 0.89–1.22) [105]. Overall, linagliptin use was not associated with significant reductions in cardiorenal outcomes. However, albuminuria progression was significantly suppressed by linagliptin compared to placebo (HR 0.86, 95% CI: 0.78–0.95, *p* = 0.03) [105]. A secondary analysis of CARMELINA showed that significant reductions in the risk of albuminuria progression were preserved across all eGFR categories (≥60, 45 to <60, 30 to <45, and <30 mL/min/1.73 m^2^) [106].

The MARLINA-T2D study was performed to investigate the glycemic and renal effects of linagliptin on T2D patients with albuminuria [107]. A total of 360 T2D patients with eGFR ≥ 30 mL/min/1.73 m^2^ and a urinary albumin-to-creatinine ratio (UACR) 30–3000 mg/g were randomized to receive linagliptin (*n* = 182) or placebo (*n* = 178) for 24 weeks. The secondary endpoint was the time-weighted average of percentage change from baseline in UACR at 24 weeks. Linagliptin significantly improved the glycemic control (−0.6% compared to placebo) but not albuminuria, with no evidence of renal adverse effects, in a high-risk population of patients with T2D who have albuminuria [107].

### 6.2. Saxagliptin

Several major clinical trials to assess cardiovascular and renal outcomes have been reported. In the Saxagliptin Assessment of Vascular Outcomes Recorded in Patients with Diabetes Mellitus–Thrombolysis in Myocardial Infarction (SAVOR-TIMI 53) study, a total of 16,492 T2D patients with a history of CVD (approximately 80% of the participants) or multiple CV risks (approximately 20%) were randomized to the saxagliptin or placebo group. The primary endpoint was a composite of cardiovascular death, myocardial infarction, and ischemic stroke. After an average follow-up of 2.1 years, a primary endpoint event occurred in 613 patients in the saxagliptin group and in 609 in the placebo group (7.3% and 7.2%, respectively, 95% CI: 0.89–1.12; *p* = 0.99 for superiority; *p* < 0.001 for noninferiority). However, the rate of hospitalization for heart failure was significantly higher in the saxagliptin group than in the placebo group (3.5% vs. 2.8%; HR, 1.27; 95% CI: 1.07–1.51, *p* = 0.007). Importantly, saxagliptin reduced albuminuria, regardless of the baseline eGFR. Although not directly related to the medicinal effect, a sub-analysis of SAVOR-TIMI53 was performed to examine the relationship between the UACR categories and CV risk and demonstrated that the UACR was independently associated with an increased risk of CVD. However, the incremental cardiovascular prognostic value of UACR was minimal [108]. At baseline, 9696 (58.8%) subjects had normoalbuminuria (UACR < 30 mg/g), 4426 (26.8%) had microalbuminuria (UACR 30–300 mg/g), and 1638 (9.9%) had macroalbuminuria (UACR > 300 mg/g). Saxagliptin use resulted in an improvement in the UACR even in the normoalbuminuric range, without affecting the eGFR. The difference in the mean UACR change between the saxagliptin and placebo groups was −19.3 mg/g for an eGFR > 50 mL/min/1.73 m^2^, −105 mg/g for 50 ≥ eGFR ≥ 30 mL/min/1.73 m^2^, and −245.2 mg/g for an eGFR < 30 mL/min/1.73 m^2^ [109]. Given that the HbA1c reduction was significant in the saxagliptin group at 2 years (7.5% in saxagliptin vs. 7.8% in placebo, *p* < 0.01) [110], whether the saxagliptin-mediated reduction of albuminuria was due to a glucose-lowering or an incretin-dependent mechanism remains unclear.

### 6.3. Sitagliptin

The Trial Evaluating Cardiovascular Outcomes with Sitagliptin (TECOS) assigned 14,671 T2D patients with CVD to add either sitagliptin or placebo to their existing therapy. Sitagliptin was noninferior to placebo with regard to 3P-MACE (HR 0.98, 95% CI: 0.88–1.09; *p* < 0.001). The rates of hospitalization for heart failure did not differ markedly between the two groups (HR, 1.00; 95% CI, 0.83–1.20; *p* = 0.98) after a median follow-up of 3.0 years. A sub-analysis of the TECOS demonstrated that the kidney function decreased at the same rate in both treatment groups, with a marginally lower but constant eGFR difference (−1.3 mL/min/1.73 m^2^) in the participants who were assigned to sitagliptin rather than placebo during the follow-up period, indicating that sitagliptin has no marked effect on the eGFR [111].

The effect on albuminuria was not reported in the TECOS. However, smaller studies have found that sitagliptin inhibits albuminuria. A single-arm study showed that the administration of sitagliptin for six months decreased the urinary albumin excretion, irrespective of the UACR category, including normoalbuminuria, along with an HbA1c reduction in 36 T2D subjects [112]. Another study including 247 T2D patients revealed that sitagliptin use for 3 months reduced the UACR. Intriguingly, the UACR reductions were not shown to be correlated with a decrease in the HbA1c over a three-month period [113]. Consistent with this observation, an RCT including 85 T2D patients showed that the administration of sitagliptin for 6 months reduced the UACR significantly compared to the control group, whereas the glycemic control was comparable in both groups [114].

### 6.4. Alogliptin

In the Examination of Cardiovascular Outcomes with Alogliptin versus Standard of Care (EXAMINE) trial, a total of 5380 T2D patients with either acute myocardial infarction or unstable angina requiring hospitalization within the previous 15 to 90 days were assigned to receive alogliptin or placebo. The primary endpoint was 3-P MACE, defined as a composite of death from cardiovascular causes, nonfatal myocardial infarction, and nonfatal stroke. After a median follow-up of 18 months, the incidence of 3-P MACE was not increased in the alogliptin group [115].

Fujita et al. investigated the effect of the combination of DPP-4 inhibitors with ARB in T2D patients with early-stage DKD. The study consisted of 3 treatment periods: sitagliptin 50 mg/day for 4 weeks (first period), alogliptin 25 mg/day for 4 weeks (second period), and sitagliptin 50 mg/day for 4 weeks (third period) [116]. Interestingly, they observed that switching from sitagliptin to alogliptin decreased albuminuria and 8-hydroxy-2′-deoxyguanosine (8-OHdG). Furthermore, it increased urinary cAMP levels and plasma levels of SDF-1α [116]. These findings suggest that alogliptin exerts renoprotective effects by inhibiting oxidative stress by preventing SDF-1α degradation by DPP-4. Furthermore, it has been reported that 12 weeks of administration of aloglitpin in T2D patients resulted in an UACR reduction along with a decrease in the AGE/RAGE axis activity [117].

### 6.5. Vildagliptin

A single-arm study enrolling 47 T2D subjects who were administered vildagliptin for 8 weeks resulted in reductions in plasma small dense low-density lipoprotein (LDL) levels (−8.8%) and the UACR (−44.6%) compared with the baseline [118]. A recent RCT demonstrated that the administration of saxagliptin and vildagliptin for 12 weeks significantly reduced the albuminuria rate by 57.9% (95% CI: 66.1–49.8%) and 55.2% (95% CI 64.9–45.4%), respectively, compared with the control group in T2D subjects. Interestingly, these observations were not associated with HbA1c changes [119].

### 6.6. Anagliptin

To date, one study examining the effects of anagliptin on albuminuria has been reported. In that study, 25 patients with T2D with DKD received anagliptin 200 mg/day, and 20 patients who switched to anagliptin from other DPP-4 inhibitors were involved. After 24 weeks, anagliptin use resulted in significant reductions in the UACR (down 10.6% from baseline) and the urinary liver-type fatty acid-binding protein to creatinine ratio (LFABP) (down 58.1% from baseline) [120]. Interestingly, significant changes in the HbA1c, lipid data, systolic blood pressure, and renal function were not observed during the study period, indicating the glucose-independent renoprotective effects of anagliptin [120].

### 6.7. Teneligliptin

A single-arm, open-label, observational study showed that switching to teneligliptin from other DPP-4 inhibitors for 24 weeks reduced the plasma DPP-4 activity, which was associated with a reduction in albuminuria, independent of the glucose-lowering effects, in T2D patients with early-stage DKD [121].

Taken together, these findings demonstrate that DPP-4 inhibitors have modest effects of reducing albuminuria but no effects of eGFR decline. The results of major clinical trials are shown in Table 1.

## 7. Meta-Analysis of DPP-4 Inhibitors on Renal Outcomes

A recent meta-analysis of RCTs including 47,955 patients demonstrated that use of DPP-4 inhibitors was associated with a greater decline in eGFR than treatment with the comparators (weighted mean difference −1.12 mL/min/1.73 m^2^, 95% CI: −1.61 to −0.62). In addition, DPP-4 inhibitors significantly reduced the risks of new onset of albuminuria (RR 0.88, 95% CI: 0.8–0.98) and progression of albuminuria (RR 0.88, 95% CI: 0.82–0.94) [122]. Consistently, another meta-analysis including 23 RCTs (41,359 patients) reported that DPP-4 inhibitors significantly reduced the risk of microalbuminuria (RR 0.89, 95% CI: 0.80–0.98) and macroalbuminuria (RR 0.77, 95% CI: 0.61–0.97), as well as higher rates of regression of albuminuria (RR: 1.22, 95% CI: 1.10–1.35) compared with controls. Furthermore, use of DPP-4 inhibitors was associated with lower eGFR (weighted mean difference, −1.11 mL/min/1.73 m^2^, 95% CI: −1.78 to −0.44) [123]. Taken together, clinical benefit of DPP-4 inhibitors on renal outcome is mainly driven by reduction of albuminuria.

## 8. Safety and Efficacy of DPP-4 Inhibitors in Patients with an Impaired Renal Function

DPP-4 inhibitors can be administered to patients with an impaired renal function by adjusting the dose. However, linaglitpin does not require dose adjustment because it is mainly excreted in the feces without any metabolic conversion by the liver, whereas most other DPP4-inhibitors are predominantly excreted via the urine [124]. A meta-analysis including six placebo/open and two active controlled trials (*n* = 1747) evaluating the safety and efficacy of DPP-4 inhibitors on T2D patients with moderate to severe renal impairment showed that the adjusted mean difference in HbA1c between DPP-4 inhibitors and placebo ranged from −0.60% to −0.42%. The odds ratio of hypoglycemia, mortality, and severe adverse effects due to all types of DPP-4 inhibitors were 1.35 (95% CI: 0.98–1.84), 0.88 (95% CI: 0.42–1.86), and 0.86 (95% CI: 0.65–1.15), respectively [125]. Accordingly, Walker et al. reported a meta-analysis evaluating the safety and efficacy of DPP-4 inhibitors in T2D patients with CKD (defined as an eGFR < 60 mL/min/1.73 m^2^) and dialysis [126]. They included 12 studies with 4403 patients with CKD and 239 on dialysis and demonstrated that a mean weighted decline in HbA1c of −0.48 (95% CI −0.61 to −0.35) with DPP-4 inhibitor therapy compared to placebo without any increased risk of hypoglycemia or mortality [126]. The efficacy and safety of omaliglitpin, a once weekly DPP-4 inhibitor, has also been reported. Patients with T2D and moderate (eGFR 30–60 mL/min/1.73 m^2^) (*n* = 114) or severe (eGFR < 30 mL/min/1.73 m^2^) (*n* = 55) or ESRD on dialysis (*n* = 44) were allocated to the placebo or omaligliptin group and followed for 24 weeks [127]. The omaligliptin group showed a marked HbA1c reduction from baseline (−0.33% compared with the placebo group) without an increased risk of adverse events, including hypoglycemia [127]. Finally, teneligliptin was shown to improve an impaired lipid metabolism by reducing the plasma level of remnant-like particle cholesterol in patients with T2D who were undergoing hemodialysis [128].

## 9. Conclusions and Perspectives

DKD is a multifactorial diabetic complication. Hyperglycemia, hypertension, and dyslipidemia play crucial roles in the development of DKD. It is well established that intensive glycemic control attenuates the onset and progression of DKD [129]. J-DOIT3 revealed that intensified multifactorial intervention can inhibit DKD [130]. In that study, 2541 T2D patients with either or both hypertension and dyslipidemia were allocated to receive intensive therapy or conventional therapy. After a median follow-up of 8.5 years, intensive therapy resulted in a 32% reduction (HR 0.68, 95% CI: 0.56–0.82; *p* < 0.0001) in DKD (defined as progression from normoalbuminuria (UACR < 30 mg/g) to microalbuminuria (UACR ≥ 30 mg/g to <300 mg/g), from normoalbuminuria to macroalbuminuria (UACR ≥ 300 mg/g), from microalbuminuria to macroalbuminuria, a ≥2-fold increase in the serum creatinine concentration compared with the study entry, or end-stage renal failure) compared with conventional therapy [130]. A sub-analysis of J-DOIT3 showed that multifactorial intervention and glycemic control reduced onset of DKD, while blood pressure control suppressed the eGFR decline [131]. In contrast, the effects of intensified multifactorial intervention on advanced DKD have not been described. Recently, the Diabetic Nephropathy Remission and Regression Team Trial in Japan (DNETT-Japan) reported a non-significant trend toward a reduction in renal events, defined as ESRD, doubling serum creatinine levels, or death (HR 0.69, 95% CI: 0.43–1.11; *p* = 0.13) [132].

As these abovementioned studies show, it is important to initiate and sustain drug intervention in the early stage of DKD. DPP-4 inhibitors can be used at any stage, regardless of the renal function, with CV safety. In this respect, DPP-4 inhibitors may be suitable drugs for preventing the progression of DKD.

Although various experimental studies have demonstrated that DPP-4 inhibitors attenuate DKD in a glucose-dependent and glucose-independent fashion, clinical studies have revealed modest effects on albuminuria. Why clinical trials have not yet revealed any significant benefits of DPP-4 inhibitors on hard renal outcomes, despite their beneficial effects on DKD shown in preclinical studies, is unclear at present but may be related to the diversification of the pathophysiology underlying renal complications with diabetes, as heterogeneity of DKD has emerged [133]. Recently, a sub-analysis of CREDENCE showed that canagliflozin significantly slowed the progression of the eGFR reduction, even in patients with an eGFR < 30 mL/min/1.73 m^2^ [134]. DPP-4 inhibitors may be inferior in terms of renal protection compared to SGLT2 inhibitors, but DPP-4 inhibitors remain valuable because they can be used regardless of the renal function and confer stable glycemic control with little risk of hypoglycemia. Furthermore, DPP-4 inhibitors have unique renoprotective mechanisms that are mediated by DPP-4 substrates and microRNAs. As DKD can be caused by multiple mechanisms, combination therapy with other anti-diabetic drugs with pleiotropic effects, such as SGLT2 inhibitors and/or metformin, may aid in synergistic renoprotection. These combination therapy may confirm whether DPP-4 inhibitors exert significant renoprotectivie effects in clinical settings. In major clinical trials of DPP-4 inhibitors, including CARMELINA, the follow-up period for assessing hard renal endpoints was relatively short. Clinical trials or observational studies with a longer follow-up period will address these issues.

## Figures and Tables

**Table 1:** Renal outcomes of major clinical trials using DPP-4 inhibitors. The beneficial effects of DPP-4 inhibitors on albuminuria but not the reduction in the estimated glomerular filtration rate are shown.

Trials	Participants	Renal Outcome
**SAVOR-TIMI 53** (saxagliptin 2.5 mg or 5 mg vs. placebo)	T2D with high CV risk (*n* = 9696), 2 years	UACR↓ (including in normal range) No effect on eGFR
**TECOS** (sitagliptin 50 mg or 100 mg vs. placebo)	T2D with high CV risk (*n* = 14,671), 3 years	No effect on eGFR
**MARLINA-T2D** (linagliptin 5 mg vs placebo)	T2D with albuminuria (*n* = 360), 24 weeks	No significant effect on UACR
**CARMELINA** (linagliptin 5 mg vs placebo)	T2D with high CV risk and renal risk defined as reduced eGFR and micro-macro albuminuria (*n* = 6991), 2.2 years	Progression of Albuminuria↓ No effects on composite renal outcomes (time to first occurrence of adjudicated death due to renal failure, ESRD, or sustained 40% or higher decrease in eGFR from baseline)

## References

[1] Waldrop G., Zhong J., Peters M., Rajagopalan S. (2016). Incretin-Based Therapy for Diabetes: What a Cardiologist Needs to Know. J. Am. Coll. Cardiol..

[2] Mulvihill E.E., Drucker D.J. (2014). Pharmacology, physiology, and mechanisms of action of dipeptidyl peptidase-4 inhibitors. Endocr. Rev..

[3] Enz N., Vliegen G., De Meester I., Jungraithmayr W. (2019). CD26/DPP4—A potential biomarker and target for cancer therapy. Pharmacol. Ther..

[4] Deacon C.F. (2019). Physiology and Pharmacology of DPP-4 in Glucose Homeostasis and the Treatment of Type 2 Diabetes. Front. Endocrinol..

[5] Seino Y., Fukushima M., Yabe D. (2010). GIP and GLP-1, the two incretin hormones: Similarities and differences. J. Diabetes Investig..

[6] Seino Y., Yabe D. (2013). Glucose-dependent insulinotropic polypeptide and glucagon-like peptide-1: Incretin actions beyond the pancreas. J. Diabetes Investig..

[7] Zhuge F., Ni Y., Nagashimada M., Nagata N., Xu L., Mukaida N., Kaneko S., Ota T. (2016). DPP-4 Inhibition by Linagliptin Attenuates Obesity-Related Inflammation and Insulin Resistance by Regulating M1/M2 Macrophage Polarization. Diabetes.

[8] Shin J., Fukuhara A., Onodera T., Yokoyama C., Otsuki M., Shimomura I. (2017). Regulation of Dipeptidyl Peptidase-4, its Substrate Chemokines, and Their Receptors in Adipose Tissue of ob/ob Mice. Horm. Metab. Res..

[9] Varin E.M., Mulvihill E.E., Beaudry J.L., Pujadas G., Fuchs S., Tanti J.F., Fazio S., Kaur K., Cao X., Baggio L.L. (2019). Circulating Levels of Soluble Dipeptidyl Peptidase-4 Are Dissociated from Inflammation and Induced by Enzymatic DPP4 Inhibition. Cell Metab..

[10] Coppolino G., Leporini C., Rivoli L., Ursini F., di Paola E.D., Cernaro V., Arturi F., Bolignano D., Russo E., De Sarro G. (2018). Exploring the effects of DPP-4 inhibitors on the kidney from the bench to clinical trials. Pharmacol. Res..

[11] Girardi A.C., Degray B.C., Nagy T., Biemesderfer D., Aronson P.S. (2001). Association of Na(+)-H(+) exchanger isoform NHE3 and dipeptidyl peptidase IV in the renal proximal tubule. J. Biol. Chem..

[12] Tiruppathi C., Miyamoto Y., Ganapathy V., Roesel R.A., Whitford G.M., Leibach F.H. (1990). Hydrolysis and transport of proline-containing peptides in renal brush-border membrane vesicles from dipeptidyl peptidase IV-positive and dipeptidyl peptidase IV-negative rat strains. J. Biol. Chem..

[13] Carraro-Lacroix L.R., Malnic G., Girardi A.C. (2009). Regulation of Na+/H+ exchanger NHE3 by glucagon-like peptide 1 receptor agonist exendin-4 in renal proximal tubule cells. Am. J. Physiol. Ren. Physiol..

[14] Schlatter P., Beglinger C., Drewe J., Gutmann H. (2007). Glucagon-like peptide 1 receptor expression in primary porcine proximal tubular cells. Regul. Pept..

[15] Pyke C., Heller R.S., Kirk R.K., Orskov C., Reedtz-Runge S., Kaastrup P., Hvelplund A., Bardram L., Calatayud D., Knudsen L.B. (2014). GLP-1 receptor localization in monkey and human tissue: Novel distribution revealed with extensively validated monoclonal antibody. Endocrinology.

[16] Ronn J., Jensen E.P., Wewer Albrechtsen N.J., Holst J.J., Sorensen C.M. (2017). Glucagon-like peptide-1 acutely affects renal blood flow and urinary flow rate in spontaneously hypertensive rats despite significantly reduced renal expression of GLP-1 receptors. Physiol. Rep..

[17] Lee J.W., Chou C.L., Knepper M.A. (2015). Deep Sequencing in Microdissected Renal Tubules Identifies Nephron Segment-Specific Transcriptomes. J. Am. Soc. Nephrol..

[18] Jensen E.P., Poulsen S.S., Kissow H., Holstein-Rathlou N.H., Deacon C.F., Jensen B.L., Holst J.J., Sorensen C.M. (2015). Activation of GLP-1 receptors on vascular smooth muscle cells reduces the autoregulatory response in afferent arterioles and increases renal blood flow. Am. J. Physiol. Ren. Physiol..

[19] Hviid A.V.R., Sorensen C.M. (2020). Glucagon-like peptide-1 receptors in the kidney: Impact on renal autoregulation. Am. J. Physiol. Ren. Physiol..

[20] Duvnjak L., Perkovic M.N., Blaslov K. (2017). Dipeptidyl peptidase-4 activity is associated with urine albumin excretion in type 1 diabetes. J. Diabetes Complicat..

[21] Zheng T., Liu Y., Qin S., Liu H., Zhang X., Zhao H. (2016). Increased plasma dipeptidyl peptidase-4 activities are associated with high prevalence of diabetic nephropathy in Chinese patients with newly diagnosed type 2 diabetes: A cross-sectional study. Diabetes Vasc. Dis. Res..

[22] Zheng T., Baskota A., Gao Y., Tian H., Yang F. (2015). Increased plasma dipeptidyl peptidase 4 activities predict new-onset microalbuminuria in association with its proinflammatory effects in Chinese without diabetes: A four-year prospective study. Nephrol. Dial. Transplant..

[23] Cho E.H., Kim S.W. (2019). Soluble Dipeptidyl Peptidase-4 Levels Are Associated with Decreased Renal Function in Patients with Type 2 Diabetes Mellitus. Diabetes Metab. J..

[24] Baggio L.L., Varin E.M., Koehler J.A., Cao X., Lokhnygina Y., Stevens S.R., Holman R.R., Drucker D.J. (2020). Plasma levels of DPP4 activity and sDPP4 are dissociated from inflammation in mice and humans. Nat. Commun..

[25] Kawanami D., Matoba K., Utsunomiya K. (2016). Signaling pathways in diabetic nephropathy. Histol. Histopathol..

[26] Kawanami D., Matoba K., Takeda Y., Nagai Y., Akamine T., Yokota T., Sango K., Utsunomiya K. (2017). SGLT2 Inhibitors as a Therapeutic Option for Diabetic Nephropathy. Int. J. Mol. Sci..

[27] Opazo-Rios L., Mas S., Marin-Royo G., Mezzano S., Gomez-Guerrero C., Moreno J.A., Egido J. (2020). Lipotoxicity and Diabetic Nephropathy: Novel Mechanistic Insights and Therapeutic Opportunities. Int. J. Mol. Sci..

[28] Nagai Y., Matoba K., Kawanami D., Takeda Y., Akamine T., Ishizawa S., Kanazawa Y., Yokota T., Utsunomiya K., Nishimura R. (2019). ROCK2 regulates TGF-beta-induced expression of CTGF and profibrotic genes via NF-kappaB and cytoskeleton dynamics in mesangial cells. Am. J. Physiol. Ren. Physiol..

[29] Matoba K., Takeda Y., Nagai Y., Kawanami D., Utsunomiya K., Nishimura R. (2019). Unraveling the Role of Inflammation in the Pathogenesis of Diabetic Kidney Disease. Int. J. Mol. Sci..

[30] Loeffler I., Wolf G. (2015). Epithelial-to-Mesenchymal Transition in Diabetic Nephropathy: Fact or Fiction?. Cells.

[31] Kumar Pasupulati A., Chitra P.S., Reddy G.B. (2016). Advanced glycation end products mediated cellular and molecular events in the pathology of diabetic nephropathy. Biomol. Concepts.

[32] Ram C., Jha A.K., Ghosh A., Gairola S., Syed A.M., Murty U.S., Naidu V.G.M., Sahu B.D. (2020). Targeting NLRP3 inflammasome as a promising approach for treatment of diabetic nephropathy: Preclinical evidences with therapeutic approaches. Eur. J. Pharmacol..

[33] Fu J., Lee K., Chuang P.Y., Liu Z., He J.C. (2015). Glomerular endothelial cell injury and cross talk in diabetic kidney disease. Am. J. Physiol. Ren. Physiol..

[34] Kanasaki K. (2016). The pathological significance of dipeptidyl peptidase-4 in endothelial cell homeostasis and kidney fibrosis. Diabetol Int..

[35] Takagaki Y., Koya D., Kanasaki K. (2017). Dipeptidyl peptidase-4 inhibition and renoprotection: The role of antifibrotic effects. Curr. Opin. Nephrol. Hypertens..

[36] Muskiet M.H.A., Tonneijck L., Smits M.M., van Baar M.J.B., Kramer M.H.H., Hoorn E.J., Joles J.A., van Raalte D.H. (2017). GLP-1 and the kidney: From physiology to pharmacology and outcomes in diabetes. Nat. Rev. Nephrol..

[37] Alicic R.Z., Cox E.J., Neumiller J.J., Tuttle K.R. (2020). Incretin drugs in diabetic kidney disease: Biological mechanisms and clinical evidence. Nat. Rev. Nephrol..

[38] Kawanami D., Takashi Y. (2020). GLP-1 Receptor Agonists in Diabetic Kidney Disease: From Clinical Outcomes to Mechanisms. Front. Pharmacol..

[39] Yin W., Jiang Y., Xu S., Wang Z., Peng L., Fang Q., Deng T., Zhao W., Zhang W., Lou J. (2019). Protein kinase C and protein kinase A are involved in the protection of recombinant human glucagon-like peptide-1 on glomeruli and tubules in diabetic rats. J. Diabetes Investig..

[40] Wang D., Luo P., Wang Y., Li W., Wang C., Sun D., Zhang R., Su T., Ma X., Zeng C. (2013). Glucagon-like peptide-1 protects against cardiac microvascular injury in diabetes via a cAMP/PKA/Rho-dependent mechanism. Diabetes.

[41] Lee Y.S., Park M.S., Choung J.S., Kim S.S., Oh H.H., Choi C.S., Ha S.Y., Kang Y., Kim Y., Jun H.S. (2012). Glucagon-like peptide-1 inhibits adipose tissue macrophage infiltration and inflammation in an obese mouse model of diabetes. Diabetologia.

[42] Kodera R., Shikata K., Kataoka H.U., Takatsuka T., Miyamoto S., Sasaki M., Kajitani N., Nishishita S., Sarai K., Hirota D. (2011). Glucagon-like peptide-1 receptor agonist ameliorates renal injury through its anti-inflammatory action without lowering blood glucose level in a rat model of type 1 diabetes. Diabetologia.

[43] Ishibashi Y., Nishino Y., Matsui T., Takeuchi M., Yamagishi S. (2011). Glucagon-like peptide-1 suppresses advanced glycation end product-induced monocyte chemoattractant protein-1 expression in mesangial cells by reducing advanced glycation end product receptor level. Metabolism.

[44] Saha S., Buttari B., Panieri E., Profumo E., Saso L. (2020). An Overview of Nrf2 Signaling Pathway and Its Role in Inflammation. Molecules.

[45] Guo K., Jin F. (2019). Dipeptidyl peptidase-4 (DPP-4) inhibitor saxagliptin alleviates lipopolysaccharide-induced acute lung injury via regulating the Nrf-2/HO-1 and NF-kappaB pathways. J. Invest. Surg..

[46] Abdel-Gaber S.A., Geddawy A., Moussa R.A. (2019). The hepatoprotective effect of sitagliptin against hepatic ischemia reperfusion-induced injury in rats involves Nrf-2/HO-1 pathway. Pharmacol. Rep..

[47] Si J., Meng R., Gao P., Hui F., Li Y., Liu X., Yang B. (2019). Linagliptin protects rat carotid artery from balloon injury and activates the NRF2 antioxidant pathway. Exp. Anim..

[48] Wang C., Li C., Peng H., Ye Z., Zhang J., Liu X., Lou T. (2014). Activation of the Nrf2-ARE pathway attenuates hyperglycemia-mediated injuries in mouse podocytes. Cell Physiol. Biochem..

[49] Civantos E., Bosch E., Ramirez E., Zhenyukh O., Egido J., Lorenzo O., Mas S. (2017). Sitagliptin ameliorates oxidative stress in experimental diabetic nephropathy by diminishing the miR-200a/Keap-1/Nrf2 antioxidant pathway. Diabetes Metab. Syndr. Obes..

[50] Feng Q., Liu D., Lu Y., Liu Z. (2020). The Interplay of Renin-Angiotensin System and Toll-Like Receptor 4 in the Inflammation of Diabetic Nephropathy. J. Immunol. Res..

[51] Ibrahim S.S.A., Salama M.A., Selima E., Shehata R.R. (2020). Sitagliptin and tofacitinib ameliorate adjuvant induced arthritis via modulating the cross talk between JAK/STAT and TLR-4/NF-kappaB signaling pathways. Life Sci..

[52] Helal M.G., Megahed N.A., Abd Elhameed A.G. (2019). Saxagliptin mitigates airway inflammation in a mouse model of acute asthma via modulation of NF-kB and TLR4. Life Sci..

[53] El-Kashef D.H., Serrya M.S. (2019). Sitagliptin ameliorates thioacetamide-induced acute liver injury via modulating TLR4/NF-KB signaling pathway in mice. Life Sci..

[54] Schultheis P.J., Clarke L.L., Meneton P., Miller M.L., Soleimani M., Gawenis L.R., Riddle T.M., Duffy J.J., Doetschman T., Wang T. (1998). Renal and intestinal absorptive defects in mice lacking the NHE3 Na+/H+ exchanger. Nat. Genet..

[55] Rieg T., Gerasimova M., Murray F., Masuda T., Tang T., Rose M., Drucker D.J., Vallon V. (2012). Natriuretic effect by exendin-4, but not the DPP-4 inhibitor alogliptin, is mediated via the GLP-1 receptor and preserved in obese type 2 diabetic mice. Am. J. Physiol. Ren. Physiol..

[56] Vallon V., Docherty N.G. (2014). Intestinal regulation of urinary sodium excretion and the pathophysiology of diabetic kidney disease: A focus on glucagon-like peptide 1 and dipeptidyl peptidase 4. Exp. Physiol..

[57] Moroi M., Kubota T. (2015). Diuretic and Natriuretic Effects of Dipeptidyl Peptidase-4 Inhibitor Teneligliptin: The Contribution of Glucagon-like Peptide-1. J. Cardiovasc. Pharmacol..

[58] Lovshin J.A., Rajasekeran H., Lytvyn Y., Lovblom L.E., Khan S., Alemu R., Locke A., Lai V., He H., Hittle L. (2017). Dipeptidyl Peptidase 4 Inhibition Stimulates Distal Tubular Natriuresis and Increases in Circulating SDF-1alpha(1-67) in Patients With Type 2 Diabetes. Diabetes Care.

[59] Kanasaki K. (2018). The role of renal dipeptidyl peptidase-4 in kidney disease: Renal effects of dipeptidyl peptidase-4 inhibitors with a focus on linagliptin. Clin. Sci..

[60] Shi S., Srivastava S.P., Kanasaki M., He J., Kitada M., Nagai T., Nitta K., Takagi S., Kanasaki K., Koya D. (2015). Interactions of DPP-4 and integrin beta1 influences endothelial-to-mesenchymal transition. Kidney Int..

[61] Khalil R., Shata A., Abd El-Kader E.M., Sharaf H., Abdo W.S., Amin N.A., Saber S. (2020). Vildagliptin, a DPP-4 inhibitor, attenuates carbon tetrachloride-induced liver fibrosis by targeting ERK1/2, p38alpha, and NF-kappaB signaling. Toxicol. Appl. Pharmacol..

[62] Aboulmagd Y.M., El-Bahy A.A.Z., Menze E.T., Azab S.S., El-Demerdash E. (2020). Role of linagliptin in preventing the pathological progression of hepatic fibrosis in high fat diet and streptozotocin-induced diabetic obese rats. Eur. J. Pharmacol..

[63] Srivastava S.P., Goodwin J.E., Kanasaki K., Koya D. (2020). Inhibition of Angiotensin-Converting Enzyme Ameliorates Renal Fibrosis by Mitigating DPP-4 Level and Restoring Antifibrotic MicroRNAs. Genes.

[64] Wahba N.S., Ghareib S.A., Abdelghany R.H., Abdel-Aal M., Alsemeh A.E. (2020). Vitamin D3 potentiates the nephroprotective effects of metformin in a rat model of metabolic syndrome: Role of AMPK/SIRT1 activation and DPP-4 inhibition. Can. J. Physiol. Pharmacol..

[65] Kawakubo M., Tanaka M., Ochi K., Watanabe A., Saka-Tanaka M., Kanamori Y., Yoshioka N., Yamashita S., Goto M., Itoh M. (2020). Dipeptidyl peptidase-4 inhibition prevents nonalcoholic steatohepatitis-associated liver fibrosis and tumor development in mice independently of its anti-diabetic effects. Sci. Rep..

[66] Soare A., Gyorfi H.A., Matei A.E., Dees C., Rauber S., Wohlfahrt T., Chen C.W., Ludolph I., Horch R.E., Bauerle T. (2020). Dipeptidylpeptidase 4 as a Marker of Activated Fibroblasts and a Potential Target for the Treatment of Fibrosis in Systemic Sclerosis. Arthritis Rheumatol..

[67] Hirose M., Takano H., Hasegawa H., Tadokoro H., Hashimoto N., Takemura G., Kobayashi Y. (2017). The effects of dipeptidyl peptidase-4 on cardiac fibrosis in pressure overload-induced heart failure. J. Pharmacol. Sci..

[68] Brown S.M., Smith C.E., Meuth A.I., Khan M., Aroor A.R., Cleeton H.M., Meininger G.A., Sowers J.R., DeMarco V.G., Chandrasekar B. (2017). Dipeptidyl Peptidase-4 Inhibition With Saxagliptin Ameliorates Angiotensin II-Induced Cardiac Diastolic Dysfunction in Male Mice. Endocrinology.

[69] Zhao J., Pei L. (2020). Cardiac Endocrinology: Heart-Derived Hormones in Physiology and Disease. JACC Basic Transl. Sci..

[70] Seki N., Matsumoto T., Fukazawa M. (2018). Relationship Between the Brain Natriuretic Peptide (BNP) Level and Prognosis of Diabetic Nephropathy with Microalbuminuria: A 7-Year Follow-Up Study. Horm. Metab. Res..

[71] Furukawa S., Sakai T., Niiya T., Miyaoka H., Miyake T., Yamamoto S., Tanaka K., Ueda T., Senba H., Torisu M. (2017). B-type natriuretic peptide and renal function in Japanese patients with type 2 diabetes mellitus: The Dogo Study. Endocr. J..

[72] Seki N., Matsumoto T., Fukazawa M. (2015). Relationship between the brain natriuretic peptide (BNP) level and remission of diabetic nephropathy with microalbuminuria: A 3-year follow-up study. Horm. Metab. Res..

[73] Semenza G.L. (1994). Regulation of erythropoietin production. New insights into molecular mechanisms of oxygen homeostasis. Hematol. Oncol. Clin. North. Am..

[74] Tsuruya K., Yoshida H., Suehiro T., Fujisaki K., Masutani K., Kitazono T. (2016). Erythropoiesis-stimulating agent slows the progression of chronic kidney disease: A possibility of a direct action of erythropoietin. Ren. Fail..

[75] Sano M., Takei M., Shiraishi Y., Suzuki Y. (2016). Increased Hematocrit During Sodium-Glucose Cotransporter 2 Inhibitor Therapy Indicates Recovery of Tubulointerstitial Function in Diabetic Kidneys. J. Clin. Med. Res..

[76] Broxmeyer H.E., Hoggatt J., O’Leary H.A., Mantel C., Chitteti B.R., Cooper S., Messina-Graham S., Hangoc G., Farag S., Rohrabaugh S.L. (2012). Dipeptidylpeptidase 4 negatively regulates colony-stimulating factor activity and stress hematopoiesis. Nat. Med..

[77] Chen W.C., Liu Y.B., Liu W.F., Zhou Y.Y., He H.F., Lin S. (2020). Neuropeptide Y Is an Immunomodulatory Factor: Direct and Indirect. Front. Immunol..

[78] Lay A.C., Barrington A.F., Hurcombe J.A., Ramnath R.D., Graham M., Lewis P.A., Wilson M.C., Heesom K.J., Butler M.J., Perrett R.M. (2020). A role for NPY-NPY2R signaling in albuminuric kidney disease. Proc. Natl. Acad. Sci. USA.

[79] Hasan A.A., Hocher B. (2017). Role of soluble and membrane-bound dipeptidyl peptidase-4 in diabetic nephropathy. J. Mol. Endocrinol..

[80] Sayyed S.G., Hagele H., Kulkarni O.P., Endlich K., Segerer S., Eulberg D., Klussmann S., Anders H.J. (2009). Podocytes produce homeostatic chemokine stromal cell-derived factor-1/CXCL12, which contributes to glomerulosclerosis, podocyte loss and albuminuria in a mouse model of type 2 diabetes. Diabetologia.

[81] Nistala R., Habibi J., Aroor A., Sowers J.R., Hayden M.R., Meuth A., Knight W., Hancock T., Klein T., DeMarco V.G. (2014). DPP4 inhibition attenuates filtration barrier injury and oxidant stress in the zucker obese rat. Obesity.

[82] Takashima S., Fujita H., Fujishima H., Shimizu T., Sato T., Morii T., Tsukiyama K., Narita T., Takahashi T., Drucker D.J. (2016). Stromal cell-derived factor-1 is upregulated by dipeptidyl peptidase-4 inhibition and has protective roles in progressive diabetic nephropathy. Kidney Int..

[83] Liu W.J., Xie S.H., Liu Y.N., Kim W., Jin H.Y., Park S.K., Shao Y.M., Park T.S. (2012). Dipeptidyl peptidase IV inhibitor attenuates kidney injury in streptozotocin-induced diabetic rats. J. Pharmacol. Exp. Ther..

[84] Kodera R., Shikata K., Takatsuka T., Oda K., Miyamoto S., Kajitani N., Hirota D., Ono T., Usui H.K., Makino H. (2014). Dipeptidyl peptidase-4 inhibitor ameliorates early renal injury through its anti-inflammatory action in a rat model of type 1 diabetes. Biochem. Biophys. Res. Commun..

[85] Vavrinec P., Henning R.H., Landheer S.W., Wang Y., Deelman L.E., Dokkum R.P., Buikema H. (2014). Vildagliptin restores renal myogenic function and attenuates renal sclerosis independently of effects on blood glucose or proteinuria in zucker diabetic fatty rat. Curr. Vasc. Pharmacol..

[86] Birnbaum Y., Bajaj M., Qian J., Ye Y. (2016). Dipeptidyl peptidase-4 inhibition by Saxagliptin prevents inflammation and renal injury by targeting the Nlrp3/ASC inflammasome. BMJ Open Diabetes Res. Care..

[87] Nakashima S., Matsui T., Takeuchi M., Yamagishi S.I. (2014). Linagliptin blocks renal damage in type 1 diabetic rats by suppressing advanced glycation end products-receptor axis. Horm. Metab. Res..

[88] Oraby M.A., El-Yamany M.F., Safar M.M., Assaf N., Ghoneim H.A. (2019). Amelioration of Early Markers of Diabetic Nephropathy by Linagliptin in Fructose-Streptozotocin-Induced Type 2 Diabetic Rats. Nephron.

[89] Spencer N.Y., Yang Z., Sullivan J.C., Klein T., Stanton R.C. (2018). Linagliptin unmasks specific antioxidant pathways protective against albuminuria and kidney hypertrophy in a mouse model of diabetes. PLoS ONE.

[90] Takagaki Y., Shi S., Katoh M., Kitada M., Kanasaki K., Koya D. (2019). Dipeptidyl peptidase-4 plays a pathogenic role in BSA-induced kidney injury in diabetic mice. Sci. Rep..

[91] Li L., Lian X., Wang Z., Zheng J., Liu J., Chu Y., Teng Y., Zhang Z. (2019). The dipeptidyl peptidase-4 inhibitor sitagliptin ameliorates renal injury in type 1 diabetic mice via inhibiting the TGF-beta/Smad signal pathway. Pharmazie.

[92] Ren X., Zhu R., Liu G., Xue F., Wang Y., Xu J., Zhang W., Yu W., Li R. (2019). Effect of sitagliptin on tubulointerstitial Wnt/beta-catenin signalling in diabetic nephropathy. Nephrology.

[93] Gangadharan Komala M., Gross S., Zaky A., Pollock C., Panchapakesan U. (2016). Saxagliptin reduces renal tubulointerstitial inflammation, hypertrophy and fibrosis in diabetes. Nephrology.

[94] Kanasaki K., Shi S., Kanasaki M., He J., Nagai T., Nakamura Y., Ishigaki Y., Kitada M., Srivastava S.P., Koya D. (2014). Linagliptin-mediated DPP-4 inhibition ameliorates kidney fibrosis in streptozotocin-induced diabetic mice by inhibiting endothelial-to-mesenchymal transition in a therapeutic regimen. Diabetes.

[95] Shi S., Kanasaki K., Koya D. (2016). Linagliptin but not Sitagliptin inhibited transforming growth factor-beta2-induced endothelial DPP-4 activity and the endothelial-mesenchymal transition. Biochem. Biophys. Res. Commun..

[96] Gavrilova Y.S., Bgatova N.P., Klimontov V.V., Ischenko I.Y., Michurina S.V., Myakina N.E., Zavyalov E.L. (2016). Effect of Linagliptin on Structural Changes in the Kidney in Experimental Type 2 Diabetes Mellitus. Bull. Exp. Biol. Med..

[97] Kubo A., Hidaka T., Nakayama M., Sasaki Y., Takagi M., Suzuki H., Suzuki Y. (2020). Protective effects of DPP-4 inhibitor on podocyte injury in glomerular diseases. BMC Nephrol..

[98] Marques C., Mega C., Goncalves A., Rodrigues-Santos P., Teixeira-Lemos E., Teixeira F., Fontes-Ribeiro C., Reis F., Fernandes R. (2014). Sitagliptin prevents inflammation and apoptotic cell death in the kidney of type 2 diabetic animals. Mediat. Inflamm..

[99] Tsuprykov O., Ando R., Reichetzeder C., von Websky K., Antonenko V., Sharkovska Y., Chaykovska L., Rahnenfuhrer J., Hasan A.A., Tammen H. (2016). The dipeptidyl peptidase inhibitor linagliptin and the angiotensin II receptor blocker telmisartan show renal benefit by different pathways in rats with 5/6 nephrectomy. Kidney Int..

[100] Helmy M.M., Mouneir S.M. (2019). Reno-protective effect of linagliptin against gentamycin nephrotoxicity in rats. Pharmacol. Rep..

[101] Cappetta D., Ciuffreda L.P., Cozzolino A., Esposito G., Scavone C., Sapio L., Naviglio S., D’Amario D., Crea F., Rossi F. (2019). Dipeptidyl Peptidase 4 Inhibition Ameliorates Chronic Kidney Disease in a Model of Salt-Dependent Hypertension. Oxid. Med. Cell. Longev..

[102] Tanaka Y., Kume S., Chin-Kanasaki M., Araki H., Araki S.I., Ugi S., Sugaya T., Uzu T., Maegawa H. (2016). Renoprotective effect of DPP-4 inhibitors against free fatty acid-bound albumin-induced renal proximal tubular cell injury. Biochem. Biophys. Res. Commun..

[103] Delic D., Wiech F., Urquhart R., Gabrielyan O., Rieber K., Rolser M., Tsuprykov O., Hasan A.A., Kramer B.K., Baum P. (2020). Linagliptin and telmisartan induced effects on renal and urinary exosomal miRNA expression in rats with 5/6 nephrectomy. Sci. Rep..

[104] Hasan A.A., von Websky K., Reichetzeder C., Tsuprykov O., Gaballa M.M.S., Guo J., Zeng S., Delic D., Tammen H., Klein T. (2019). Mechanisms of GLP-1 receptor-independent renoprotective effects of the dipeptidyl peptidase type 4 inhibitor linagliptin in GLP-1 receptor knockout mice with 5/6 nephrectomy. Kidney Int..

[105] Rosenstock J., Perkovic V., Johansen O.E., Cooper M.E., Kahn S.E., Marx N., Alexander J.H., Pencina M., Toto R.D., Wanner C. (2019). Effect of Linagliptin vs Placebo on Major Cardiovascular Events in Adults With Type 2 Diabetes and High Cardiovascular and Renal Risk: The CARMELINA Randomized Clinical Trial. JAMA.

[106] Perkovic V., Toto R., Cooper M.E., Mann J.F.E., Rosenstock J., McGuire D.K., Kahn S.E., Marx N., Alexander J.H., Zinman B. (2020). Effects of Linagliptin on Cardiovascular and Kidney Outcomes in People With Normal and Reduced Kidney Function: Secondary Analysis of the CARMELINA Randomized Trial. Diabetes Care.

[107] Groop P.H., Cooper M.E., Perkovic V., Hocher B., Kanasaki K., Haneda M., Schernthaner G., Sharma K., Stanton R.C., Toto R. (2017). Linagliptin and its effects on hyperglycaemia and albuminuria in patients with type 2 diabetes and renal dysfunction: The randomized MARLINA-T2D trial. Diabetes Obes. Metab..

[108] Scirica B.M., Mosenzon O., Bhatt D.L., Udell J.A., Steg P.G., McGuire D.K., Im K., Kanevsky E., Stahre C., Sjostrand M. (2018). Cardiovascular Outcomes According to Urinary Albumin and Kidney Disease in Patients With Type 2 Diabetes at High Cardiovascular Risk: Observations From the SAVOR-TIMI 53 Trial. JAMA Cardiol..

[109] Mosenzon O., Leibowitz G., Bhatt D.L., Cahn A., Hirshberg B., Wei C., Im K., Rozenberg A., Yanuv I., Stahre C. (2017). Effect of Saxagliptin on Renal Outcomes in the SAVOR-TIMI 53 Trial. Diabetes Care.

[110] Scirica B.M., Bhatt D.L., Braunwald E., Steg P.G., Davidson J., Hirshberg B., Ohman P., Frederich R., Wiviott S.D., Hoffman E.B. (2013). Saxagliptin and cardiovascular outcomes in patients with type 2 diabetes mellitus. N. Engl. J. Med..

[111] Cornel J.H., Bakris G.L., Stevens S.R., Alvarsson M., Bax W.A., Chuang L.M., Engel S.S., Lopes R.D., McGuire D.K., Riefflin A. (2016). Effect of Sitagliptin on Kidney Function and Respective Cardiovascular Outcomes in Type 2 Diabetes: Outcomes From TECOS. Diabetes Care.

[112] Hattori S. (2011). Sitagliptin reduces albuminuria in patients with type 2 diabetes. Endocr. J..

[113] Kawasaki I., Hiura Y., Tamai A., Yoshida Y., Yakusiji Y., Ikuno Y., Okada M., Ueno H., Tanaka N., Yamagami K. (2015). Sitagliptin reduces the urine albumin-to-creatinine ratio in type 2 diabetes through decreasing both blood pressure and estimated glomerular filtration rate. J. Diabetes.

[114] Mori H., Okada Y., Arao T., Tanaka Y. (2014). Sitagliptin improves albuminuria in patients with type 2 diabetes mellitus. J. Diabetes Investig..

[115] White W.B., Cannon C.P., Heller S.R., Nissen S.E., Bergenstal R.M., Bakris G.L., Perez A.T., Fleck P.R., Mehta C.R., Kupfer S. (2013). Alogliptin after acute coronary syndrome in patients with type 2 diabetes. N. Engl. J. Med..

[116] Fujita H., Taniai H., Murayama H., Ohshiro H., Hayashi H., Sato S., Kikuchi N., Komatsu T., Komatsu K., Komatsu K. (2014). DPP-4 inhibition with alogliptin on top of angiotensin II type 1 receptor blockade ameliorates albuminuria via up-regulation of SDF-1alpha in type 2 diabetic patients with incipient nephropathy. Endocr. J..

[117] Sakata K., Hayakawa M., Yano Y., Tamaki N., Yokota N., Eto T., Watanabe R., Hirayama N., Matsuo T., Kuroki K. (2013). Efficacy of alogliptin, a dipeptidyl peptidase-4 inhibitor, on glucose parameters, the activity of the advanced glycation end product (AGE)—Receptor for AGE (RAGE) axis and albuminuria in Japanese type 2 diabetes. Diabetes Metab. Res. Rev..

[118] Tani S., Nagao K., Hirayama A. (2013). Association between urinary albumin excretion and low-density lipoprotein heterogeneity following treatment of type 2 diabetes patients with the dipeptidyl peptidase-4 inhibitor, vildagliptin: A pilot study. Am. J. Cardiovasc. Drugs.

[119] Mohsen M., Elberry A.A., Mohamed Rabea A., Abdelrahim M.E.A., Hussein R.R.S. (2020). Saxagliptin and vildagliptin lowered albuminuria in patients with diabetes and hypertension independent on glycaemic control. Int. J. Clin. Pract..

[120] Kitada M., Tsuda S.I., Konishi K., Takeda-Watanabe A., Fujii M., Kanasaki K., Nishizawa M., Nakagawa A., Koya D. (2017). Anagliptin ameliorates albuminuria and urinary liver-type fatty acid-binding protein excretion in patients with type 2 diabetes with nephropathy in a glucose-lowering-independent manner. BMJ Open Diabetes Res. Care..

[121] Kitada M., Ogura Y., Nitta K., Fujii M., Kanasaki K., Konishi K., Iida Y., Nakagawa A., Koya D. (2019). Effect of switching to teneligliptin from other dipeptidyl peptidase-4 inhibitors on glucose control and renoprotection in type 2 diabetes patients with diabetic kidney disease. J. Diabetes Investig..

[122] O’Hara D.V., Parkhill T.R., Badve S.V., Jun M., Jardine M.J., Perkovic V. (2020). The effects of dipeptidyl peptidase-4 inhibitors on kidney outcomes. Diabetes Obes. Metab..

[123] Bae J.H., Kim S., Park E.G., Kim S.G., Hahn S., Kim N.H. (2019). Effects of Dipeptidyl Peptidase-4 Inhibitors on Renal Outcomes in Patients with Type 2 Diabetes: A Systematic Review and Meta-Analysis. Endocrinol. Metab..

[124] Hanssen N.M., Jandeleit-Dahm K.A. (2019). Dipeptidyl peptidase-4 inhibitors and cardiovascular and renal disease in type 2 diabetes: What have we learned from the CARMELINA trial?. Diabetes Vasc. Dis. Res..

[125] Kamiya H. (2017). A systematic review of the benefits and harms of dipeptidyl peptidase-4 inhibitor for chronic kidney disease. Hemodial. Int..

[126] Walker S.R., Komenda P., Khojah S., Al-Tuwaijri W., MacDonald K., Hiebert B., Tangri N., Nadurak S.W.D., Ferguson T.W., Rigatto C. (2017). Dipeptidyl Peptidase-4 Inhibitors in Chronic Kidney Disease: A Systematic Review of Randomized Clinical Trials. Nephron.

[127] Chacra A., Gantz I., Mendizabal G., Durlach L., O’Neill E.A., Zimmer Z., Suryawanshi S., Engel S.S., Lai E. (2017). A randomised, double-blind, trial of the safety and efficacy of omarigliptin (a once-weekly DPP-4 inhibitor) in subjects with type 2 diabetes and renal impairment. Int. J. Clin. Pract..

[128] Homma K., Yoshizawa J., Shiina Y., Ozawa H., Igarashi M., Matsuoka T., Sasaki J., Yoshizawa M., Homma Y. (2017). A Dipeptidyl Peptidase-4 Inhibitor, Teneligliptin, Decreases Plasma Triglyceride-Rich Lipoprotein Remnants in Diabetic Patients with Chronic Kidney Disease Undergoing Hemodialysis. Drugs R&D.

[129] Coca S.G., Ismail-Beigi F., Haq N., Krumholz H.M., Parikh C.R. (2012). Role of intensive glucose control in development of renal end points in type 2 diabetes mellitus: Systematic review and meta-analysis intensive glucose control in type 2 diabetes. Arch. Intern. Med..

[130] Ueki K., Sasako T., Okazaki Y., Kato M., Okahata S., Katsuyama H., Haraguchi M., Morita A., Ohashi K., Hara K. (2017). Effect of an intensified multifactorial intervention on cardiovascular outcomes and mortality in type 2 diabetes (J-DOIT3): An open-label, randomised controlled trial. Lancet Diabetes Endocrinol..

[131] Ueki K., Sasako T., Okazaki Y., Miyake K., Nangaku M., Ohashi Y., Noda M., Kadowaki T., Group J.D.S. (2020). Multifactorial intervention has a significant effect on diabetic kidney disease in patients with type 2 diabetes. Kidney Int..

[132] Shikata K., Haneda M., Ninomiya T., Koya D., Suzuki Y., Suzuki D., Ishida H., Akai H., Tomino Y., Uzu T. (2020). Randomized trial of an intensified, multifactorial intervention in patients with advanced-stage diabetic kidney disease: Diabetic Nephropathy Remission and Regression Team Trial in Japan (DNETT-Japan). J. Diabetes Investig..

[133] Yamanouchi M., Furuichi K., Hoshino J., Ubara Y., Wada T. (2020). Nonproteinuric diabetic kidney disease. Clin. Exp. Nephrol..

[134] Bakris G., Oshima M., Mahaffey K.W., Agarwal R., Cannon C.P., Capuano G., Charytan D.M., de Zeeuw D., Edwards R., Greene T. (2020). Effects of Canagliflozin in Patients with Baseline eGFR <30 mL/min per 1.73 m(2): Subgroup Analysis of the Randomized CREDENCE Trial. Clin. J. Am. Soc. Nephrol..

